# Functional Categories of Visuomotor Neurons in Macaque Frontal Eye Field

**DOI:** 10.1523/ENEURO.0131-18.2018

**Published:** 2018-10-17

**Authors:** Kaleb A. Lowe, Jeffrey D. Schall

**Affiliations:** Department of Psychology, Center for Integrative and Cognitive Neuroscience, Vanderbilt Vision Research Center, Vanderbilt University, Nashville, Tennessee 37240

## Abstract

Frontal eye field (FEF) in macaque monkeys contributes to visual attention, visual–motor transformations and production of eye movements. Traditionally, neurons in FEF have been classified by the magnitude of increased discharge rates following visual stimulus presentation, during a waiting period, and associated with eye movement production. However, considerable heterogeneity remains within the traditional visual, visuomovement, and movement categories. Cluster analysis is a data-driven method of identifying self-segregating groups within a dataset. Because many cluster analysis techniques exist and outcomes vary with analysis assumptions, consensus clustering aggregates over multiple analyses, identifying robust groups. To describe more comprehensively the neuronal composition of FEF, we applied a consensus clustering technique for unsupervised categorization of patterns of spike rate modulation measured during a memory-guided saccade task. We report 10 functional categories, expanding on the traditional 3 categories. Categories were distinguished by latency, magnitude, and sign of visual response; the presence of sustained activity; and the dynamics, magnitude and sign of saccade-related modulation. Consensus clustering can include other metrics and can be applied to datasets from other brain regions to provide better information guiding microcircuit models of cortical function.

## Visual Abstract

**Figure:**
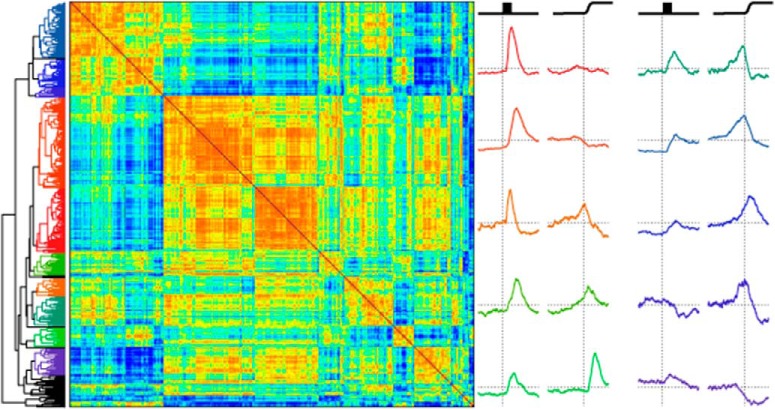

## Significance Statement

The contribution of a brain region cannot be understood without knowing the diversity, arrangement, and circuitry of constituent neurons. Traditional descriptions of frontal eye field include visual, visuosaccadic, and saccadic categories. Here, we use a novel consensus clustering method to identify more reliably functional categories in neural data. While confirming the traditional categories, consensus clustering distinguishes additional, previously unappreciated diversity in neural activity patterns. Such information is necessary to formulate correct microcircuit models of cortical function.

## Introduction

Like all cortical areas, frontal eye field (FEF) is composed of neurons distinguished by morphology, neurochemistry, biophysics, layer, and connectivity. Biophysical distinctions can be made via action potential waveforms (McCormick et al., 1985; Mitchell et al., 2007; Cohen et al., 2009a; Ding and Gold, 2012; Thiele et al., 2016), calcium binding proteins (Pouget et al., 2009), and neuromodulatory receptors (Noudoost and Moore, 2011; Soltani et al., 2013). Neurons with distinct biophysical characteristics must play different roles in the cortical microcircuit (Lewis and Lund, 1993; DeFelipe, 1997; Pouget et al., 2009; Zaitsev et al., 2012). Connectivity studies find FEF connected with at least 80 cortical areas (Huerta et al., 1986, 1987; Schall et al., 1993, 1995a; Stanton et al., 1993, 1995; Markov et al., 2014), and most pyramidal neurons do not project to more than one cortical area (Markov et al., 2014; Ninomiya et al., 2012; Pouget et al., 2009). Numerous functional distinctions among FEF neurons have been reported, beginning with the traditional sorting into visual, visuomovement, and movement plus fixation and postsaccadic categories (Bruce and Goldberg, 1985; Schall, 1991). Subsequently, FEF neurons have been implicated in numerous functions including visual search (Schall et al., 1995b; Thompson et al., 1996; Lee and Keller, 2008; Zhou and Desimone, 2011; Purcell et al., 2012a; Fernandes et al., 2014; Costello et al., 2016), saccade preparation and inhibition (Hanes et al., 1998; Boucher et al., 2007; Ray et al., 2009), perceptual choice (Ding and Gold, 2012), visual attention (Bichot et al., 1996; Bichot and Schall, 2002; Gregoriou et al., 2009; Khayat et al., 2009; Zhou and Desimone, 2011; Schafer and Moore 2011; Noudoost et al., 2014; Thiele et al., 2016), visual working memory (Clark et al., 2012; Reinhart et al., 2012), trans-saccadic stability (Crapse and Sommer, 2008, 2012; Shin and Sommer, 2012; Joiner et al., 2013; Chen et al., 2018), planning saccade sequences (Phillips and Segraves, 2010), eye–head coordination (Elsley et al., 2007; Knight 2012; Sajad et al., 2015; Izawa and Suzuki, 2018), and anticipating reward (Roesch and Olson, 2003; Glaser et al., 2016). Can so many functions be accomplished by so few neuron categories?

The problem of classification is neither new to science nor unique to neurophysiology. Cluster analysis is a powerful statistical tool, which was developed to find self-segregating categories in gene expression (Sharp et al., 1986), psychiatric diagnostics (Lochner et al., 2005), linguistics (Gries and Stefanowitsch, 2010), and Scotch whisky (Lapointe and Legendre, 1994). It has also been used to describe the biophysical diversity of cortical neurons (Nowak et al., 2003; Druckmann et al., 2013; Ardid et al., 2015), expanding the *in vivo* description of putative excitatory and inhibitory cells. Cluster analysis should be similarly powerful for assessing the functional diversity that must parallel anatomic diversity and should reproduce the functional categories known to exist in FEF.

Cluster analysis requires strategic decisions about the method of grouping observations and how to calculate pairwise distance, which lacks rigorous specification for clustering the functional characteristics of neurons. Therefore, we applied multiple preprocessing pipelines to a large sample of FEF neurons then applied an agglomerative clustering algorithm to discover functional categories. Because a priori endorsement of any particular preprocessing pipeline is impossible, and each result is unique, the results of an individual clustering procedure are difficult to interpret. However, second-order clustering procedures known as consensus clustering combine outcomes from different pipelines (Strehl and Ghosh, 2002). Distinct consensus clustering procedures use different theoretical motivations and computational efficiencies (Goder and Filkov, 2008). We applied a procedure that operates on the median pairwise similarity across all preprocessing pipelines because it is tractable and efficient. This consensus clustering procedure identified 10 robust functional categories of FEF neurons, which elaborate conventional functional classifications.

## Materials and Methods

### Subjects and behavioral task

Three male macaque monkeys (*Macaca radiata*) participated in this study. All procedures were performed in accordance with the National Institutes of Health *Guide for the Care and Use of Laboratory Animals* and were approved by the Vanderbilt Institutional Animal Care and Use Committee. Monkeys were trained to perform a memory-guided saccade task (Bruce and Goldberg, 1985). Trials began when a central fixation point appeared. After fixating this point for 500 ms, a peripheral target was presented for one screen refresh (16.7 ms at 60 Hz refresh rate) at 8° eccentricity at one of eight locations separated by 45°. After a variable delay between 300 and 800 ms the fixation point was extinguished, and the monkey was required to shift gaze to and maintain fixation on the location of the peripheral stimulus. The peripheral stimulus was reilluminated after the saccade to provide a fixation stimulus. Fluid reward was delivered if the monkey maintained fixation on the peripheral, now reilluminated, stimulus for 500 ms. If the monkey broke fixation, made a saccade to an incorrect location, or made a saccade before the fixation point was extinguished, a 5000 ms time-out delay occurred.

### Recording techniques

**Figure 1.**
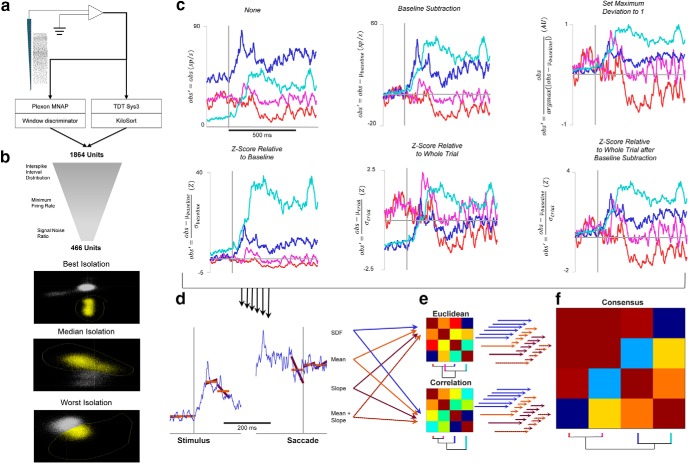
Analysis pipeline. ***a***, Potential neurons were recorded from FEF using multicontact electrode arrays. These recordings were performed either in the Plexon MNAP or the Tucker-Davis Technologies System 3. Potential units from the Plexon MNAP were sorted on-line with a window discriminator, whereas potential units from TDT Sys3 were sorted off-line using KiloSort (Pachitariu et al., 2016). A total of 1884 potential units were recorded. ***b***, The 1884 potential units were subjected to several criteria to ensure that only single units were analyzed further. These criteria include interspike interval distributions, a minimum baseline firing rate, and a signal-to-noise ratio of sorted action potential waveforms. The quality of isolation is illustrated, where the PCA space of off-line sorting is shown for the units with the best, median, and worst signal-to-noise ratio that still meet the criterion. ***c***, Six methods of scaling spike density functions were applied for normalization. Four units were selected to illustrate the effects of these different scaling methods. The colors of each unit were assigned arbitrarily. The equations for each scaling method are shown on the ordinates. Zero points in scaling and time are shown in light gray. ***d***, After each scaling method, features for inclusion in the clustering algorithm are measured. Four ways of measurement were used and are demonstrated on one of the example units from above: the full SDF (blue), the mean of the SDF during epochs of interest (orange), the slope of the SDF during epochs of interest (purple), and the combination of mean and slope. Each of these four measurements, for each of the six scaling methods, were clustered individually. ***e***, Clustering on the feature vectors generated from the scaling and measurement techniques can be performed using either Euclidean or correlation distance. Euclidean distance measures whether pairs of units have similar values of the measurements, regardless of the patterns of modulation, whereas correlation distance measures the similarity of modulation patterns regardless of absolute similarity. An example clustering dendrogram and distance matrix for each distance metric is shown as applied to the four example units, and it can be seen that these two clustering methods produce different categorizations. ***f***, Because there is no a priori way to select which scaling method, measurement, or distance metric is most appropriate, and each may produce different categorizations, the final categorization was selected by applying consensus clustering. The distance matrices for each scaling method, measurement, and distance metric (48 total combinations) were normalized and combined to create a consensus distance matrix. The same clustering algorithm was applied to this consensus distance matrix. The consensus distance matrix and corresponding final dendrogram for the four example units is shown. Final categories were determined by applying additional criteria (minimum category membership and maximum number of uncategorized neurons).

MRI-compatible headposts and recording chambers were placed over the arcuate sulcus. Surgery was conducted under aseptic conditions with animals under isoflurane anesthesia. Antibiotics and analgesics were administered postoperatively. Details have been described previously (Schall et al., 1995b; Sato et al., 2001; Cohen et al., 2009b). Data were streamed to a data acquisition system: Multi-Neuron Acquisition Processor (40 kHz; monkeys Da, Ga, and He, Plexon) or TDT System 3 (25 kHz; monkey Da, Tucker-Davis Technologies; Fig. 1*a*). Eye position was collected using EyeLink 1000 (SR Research). Eye position was calibrated daily, streamed to the data acquisition system, and stored at 1 kHz. Electrophysiological data were obtained from linear electrode arrays, either a 24-channel Plexon Uprobe (monkeys Ga and He) or a 32-channel NeuroNexus Vector Array (monkey Da). Both probes had a 150 μm recording contact spacing. Single units were identified on-line using a window discriminator (Plexon) or principle component analysis (Tucker-Davis Technologies). Units recorded from the TDT system were sorted off-line using Kilosort (Pachitariu et al., 2016).

Unit isolation was assessed by measuring waveform signal-to-noise ratio (SNR), interspike interval distributions, and baseline firing rate. SNR was calculated by dividing the voltage difference between the peak and trough of the mean action potential waveform by the Standard Deviation (SD) of concatenated waveform residuals (Joshua et al., 2007). A minimum SNR criterion was set for each recording system. Units with >10% of interspike intervals <2 ms were excluded. Units with a mean baseline discharge rate of <5 spikes/s were excluded. Of 1864 potential single units, 963 were excluded based on SNR, 22 were excluded based on interspike interval distribution, and 439 were excluded based on baseline firing rate. All together, these criteria excluded 1383 potential units, leaving 481 for analysis. An additional 15 units were excluded for lacking nonzero values in either the visual or perisaccadic epochs of their spike density function (SDF). These very conservative criteria resulted in only 25% of potential units being included in the categorization, which indicates that they were well isolated single units (Fig. 1*b*).

### Neuron classification

SDFs were calculated by convolving the spike trains with a function that resembles the postsynaptic influence of each spike (Thompson et al., 1996). SDFs were calculated only for correct trials on which the visual stimulus was presented in the visual receptive field, and the saccade was made into the movement field. The number of trials contributing to characterizing each neuron ranged from as few as 4 to as many as 317 (median, 34 trials), with trials with fewer than five spikes excluded. If the spike density function was not a stable estimate, we did not include the neuron. A sequence of classification procedures was used. The first was based on the traditional criteria of Bruce and Goldberg (1985). A unit was considered to have visual activity if the firing rate between 50 and 150 ms after stimulus presentation was elevated >6 SDs above the baseline mean. A unit was considered to have movement activity if the firing rate in the 100 ms preceding the saccade was >6 SDs above the baseline mean and the SDF showed a positive correlation over time in the 20 ms preceding saccade. This prevents elevated delay activity with no presaccadic ramping from being considered movement-related activity. Visual units had visual responses with no movement activity, movement units had movement activity with no visual responses, and visuomovement units had both. Other units were considered uncategorized; we did not test for fixation or postsaccadic activity in this categorization.

Units were categorized via agglomerative hierarchical cluster analyses (Sokal and Michener, 1958; Everitt et al., 2011). These analyses iteratively combine units, or groups of units, based on the weighted average similarity of units. In each case, the analysis algorithm was identical, although the method for determining similarity differed due to the scaling of discharge rates across units (Fig. 1*c*), measurement of the response of the units (Fig. 1*d*), or the similarity metric (Fig. 1*e*). The agglomerative cluster analysis was performed as follows: first, the sample was considered as *n* groups, each with one member. Then, the two groups with the smallest pairwise distance were combined into one group, leaving (*n* − 1) groups, one of which had two members. The distances of this group to the other groups were determined by the weighted mean of the distances of the individuals in each group, as follows Eqn. 1:

(1) $$D'(I,J)=\frac{\sum_{x\in I}\sum_{y\in J}D(x,y)+\sum_{y\in J}\sum_{x\in I}D(x,y)}{2*\left(n_{I}+n_{J}\right)},$$

where *I* is the first group in consideration and *J* is the second, *x* are the members of group *I*, *y* are the members of group *J*, and *n_I_* and *n_J_* are the number of members in groups *I* and *J*, respectively. The value of 2 in the denominator is required because the distances are symmetrical and thus represented twice in the numerator of the equation. More simply, this averages the pairwise distances of the members of *I* and *J* such that the similarity of two groups will not be skewed by uneven group sizes.

This procedure was repeated until all observations were agglomerated into a single group. Then, category identifications were made for a range of number of categories, *k,* by finding the most recent step in the algorithm at which *k* categories with a minimum of *x* members were present. For example, for a *k* value of five, the most recent set with five categories of at least *x* members was assigned as the final classification. Category membership for *k* was assessed between 1 and 20. The value of *x* was set to 10; only categories with ≥10 members were considered to assure robust results.

Category membership was assessed for six scaling procedures, four measurements of the response, and two similarity metrics (Fig. 1*c–e*). The mean skewness across time points was used to assess the quality of scaling for cross-unit comparisons. We refer to each combination of scaling procedure, SDF measurement, and similarity metric as a preprocessing pipeline. We evaluated categories derived from each pipeline but will show outcomes for only three.

Modulation of discharge rates was measured according to the following approaches. Three (mean, slope, and mean and slope) account for the firing rates during epochs: −200 to −100 ms before stimulus onset; 50–100 ms poststimulus; 100–150 ms poststimulus, −100 to −50 ms before saccade; −50 to 0 ms before saccade; and 50–100 ms postsaccade. The “mean” measurement was based on the mean firing rates in these epochs, the “slope” measurement was based on the slope of the firing rate changes in these epochs (i.e., the difference in mean firing rate at the beginning and end of the epoch), and “mean and slopes” were based on the concatenation of mean firing rate and slope. The “SDF” measurement did not parse the responses into epochs, and was instead based on the values of the SDF aligned on stimulus onset (−200 to 300 ms) or saccade (−300 to 200 ms) at each time point to millisecond resolution. To emphasize equally all epochs, means, and slopes, each measurement value was individually converted to a *z* score across the sample.

Pairwise distance was measured in the following two ways: Euclidean distance and correlation. Euclidean distance can be conceptualized as the physical distance between two points in multidimensional space while disregarding which dimensions contribute to this distance. Correlation assesses the relationships between the dimensions while disregarding the particular values of those dimensions. The different emphases of these two distance metrics can and often do assign two units to the same category via one metric but not the other.

### Euclidean distance

Based on the firing rate each unit was placed in a multidimensional space. This multidimensional space had 6 (for mean measurement and slope measurement), 12 (for mean and slope measurement), or 1002 (for SDF measurement) dimensions. The Euclidean distance between the units in this space defines a pairwise distance matrix, as follows Eqn. 2:

(2) $$D(x,y)=\sqrt{\sum_{i}^{e}{\left(x_{i}-y_{i}\right)}^{2}},$$

where *e* is the number of epochs (or milliseconds).

### Correlation

Based on the firing rate, each unit was defined as a 6-, 12-, or 1002-element vector, depending on the measurement method, and the correlation between two vectors Eqn. 3:

(3) D(x,y)=1-ρ(x,y),

measures the similarity of the modulation patterns of two units while disregarding absolute differences in firing rates.

All of these preprocessing pipelines were tested, and all produced unique results. Some pipelines produced categories that were subjectively easy to endorse, while others produced subjectively poor categories. The results of three representative pipelines are presented, two that produced poor categorizations, for different reasons, and one that produced good categorizations. The first combination scales by *z* scoring relative to baseline and mean measurement, and uses Euclidean distance. The second combination does not scale the data before measurement, uses mean measurement, and uses correlation distance. The third combination scales by *z* scoring relative to the whole trial, and mean and slope measurement.

### Consensus clustering

No *a priori* reason endorses the categories yielded by a particular SDF scaling procedure, summary of response modulation, or distance measurement. Therefore, we used a novel strategy of combining the results of multiple clustering pipelines. This algorithm considers the pairwise distance between units for each individual preprocessing pipeline tested by creating a composite distance matrix. Each individual distance matrix was *z* scored internally to correct for the absolute scale differences between different scaling procedures and distance measurements. Then, the median for each pair was selected to prevent skewing by nonoptimal pipelines. Thus, if the pairwise distance between two units was consistently small, then this composite measure was also small, whereas if the pairwise distance between two units was small in some processing pipelines but was generally larger, then the composite distance metric reflected the trend toward differences but accounted for the isolated cases of similarity. After creating this composite distance matrix, the same agglomerative algorithm used for each individual pipeline was applied to identify categories (Fig. 1*f*). Intuitively, this method was intended to distinguish units that were clustered together regardless of preprocessing pipeline from units that were members of different clusters regardless of preprocessing pipeline.

In essence, this procedure performs a clustering that operates on a distance matrix whose entries represent robustness of categorization across a number of individual procedures, or a “consensus clustering” problem (Goder and Filkov, 2008). Indeed, consensus clustering has been used to identify biophysical classes of neurons (Ardid et al., 2015). However, while conceptually similar, the previous “meta-clustering” and the present consensus clustering differ operationally in both the algorithm used for performing clustering and the preprocessing of input data. Ardid et al. (2015) performed K-means clustering, which does not provide a unique clustering solution and is highly sensitive to starting points (Bradley and Fayyad, 1998; Peña et al., 1999; Celebi and Kingravi, 2012), so their meta-clustering involved multiple iterations of the K-means procedure using the same input data and then assigning clusters via robust comembership across each iteration. Unlike Ardid et al. (2015), we used agglomerative clustering, which delivers unique solutions because no optimization steps are involved. However, we found that clustering outcomes were sensitive to the preprocessing pipeline, varying with the discharge rate scaling procedure, measurement, and distance metric. Hence, our consensus clustering approach was conceived to assess cluster assignment consistency over preprocessing steps, not local solutions to clustering one set of preprocessed data.

### Assessing number of categories

The number of categories in individual clustering procedures was selected using a lenient version of the gap procedure of Tibshirani et al. (2001), which assesses the reduction in intracluster distance with respect to randomized null sets created with no intrinsic clustering. Valid splitting of clusters should have a greater than chance reduction in intracluster distance, assessed by the SDs of the intracluster distance in the null sets, whereas excessive splitting should have a reduction in intracluster distance within the SD of the null set. The strict version selects *k* categories as the first number of categories meeting this criterion. In the lenient version of this test, each occasion on which the above criterion is met was treated as potentially valid, and visual inspection was used to determine whether categorizations were either insufficient or excessive. In some cases, due to the difficulty in creating a reasonable null-set from physiologic data, categories were selected based on the properties of the gap curve. When a reasonable null-set could not be determined, an inflection in the gap curve was identified. This inflection identified the number of categories at which the reduction of intracluster distance was markedly less than that of the previous sequence of clusters.

For consensus categories and the composite distance matrix, the means for creating a null-set to use the gap procedure of Tibshirani et al. (2001) is unclear. Therefore, in such cases a pair of criteria for determining the maximum number of categories was set: not >10% of units was allowed to remain uncategorized, and each category required at least 10 members. The maximum number of categories that met both criteria was selected.

### Comparing categorization schemes

The quality of alternative categorization schemes was assessed by calculating an index of member variability through the ratio of variances of the spike density values. Specifically, for each time point in the spike density functions, the within-category variance at that time point was divided by the variance of the category mean across time points. For each category, the average ratio was calculated, and then the grand average was taken. Because this ratio will decrease by definition as more categories are formed, a penalty for oversplitting was imposed by multiplying the grand average ratio by the square root of the number of categories. That is, for a given category the modulation strength was calculated as follows Eqn. 4:

(4) $${MS}_{c}=\frac{\sum_{t}{\left({\bar{X}}_{c,t}-{\bar{X}}_{c,.}\right)}^{2}}{N_{t}},$$

where *c* indexes category and *t*, time. Then, for each time point a ratio of variances (*RoV*) was calculated as follows Eqn. 5:

(5) RoVc,t=∑iϵc(Xi,t-X¯c,t)2NiMSc.

These are then combined by averaging the values for each category over time, then across categories, then by applying a penalty for overclustering proportional to the square root of the number of categories identified as follows Eqn. 6:

(6) $$RoV=\sqrt{N_{c}}\frac{\sum_{c}\frac{\sum_{t}{RoV}_{c,t}}{N_{t}}}{N_{c}}.$$

Small *RoV* values can be obtained either through large groupwise modulations over time or a lack of variability among the constituent members of the categories, whereas large *RoV* values are obtained through weak categorywise modulations or large variability. That is, smaller *RoV* values indicate better categorization, and vice versa. No benchmarks have been established for this index, so we interpret relative values in comparing the quality of two categorization schemes.

The similarity of two categorization schemes was assessed as follows by the Adjusted Rand Index (*ARI*; Hubert and Arabie, 1985) Eqn. 7:

(7) ARI=∑i∑jnij2-∑iai2∑jbj2/n212∑iai2+∑jbj2-∑iai2∑jbj2/n2,

where *a_i_* and *b_j_* are the counts of category *i* or *j* in categorization procedure *a* or *b*, respectively, and *n_i,j_* is the number of observations in both category *i* in categorization scheme *a* and in category *j* in categorization scheme *b*. This quantity measures the similarity of two data categorizations and is adjusted by chance cocategorization produced through the two schemes. To assess significance, each categorization was randomly shuffled separately, destroying internal structure between the two schemes, and *ARI* was recalculated. This was repeated 1000 times and *p* was the proportion of shuffled *ARI* that exceeded the nonshuffled *ARI*.

;To visualize the overlap, for each pairwise categorization combination a signed *χ^2^* was calculated as follows Eqn. 8:

(8) $${Signed\chi^{2}}_{i,j}=\frac{n_{ij}-n(\frac{n_{i}}{n}*\frac{n_{j}}{n})}{n(\frac{n_{i}}{n}*\frac{n_{j}}{n})}.$$

That is, the difference between the observed pairwise count and the count expected by the marginal probabilities of each individual categorization scheme was calculated, then normalized by that expected value. Each category assignment was shuffled separately 1000 times, and the signed χ^2^ was recomputed for each combination and iteration. A bootstrapped *z* score was then calculated for each category combination.

### Biophysical characteristics

Several biophysical characteristics were calculated for each neuron to assess the identification of consensus categories with measurements that were not included in the clustering process. The spike width of the average waveform of each neuron was calculated as the time between the initial trough of the action potential and the following peak, or the time between the initial peak and the following trough for spikes with positive deflections. Coefficient of variation (CV), which measures firing rate variability, and local coefficient of variation (CV2), which measures CV across smaller time periods, were calculated as described previously (Holt et al., 1996). Local variation (LV), which is another metric of local firing rate variability, and revised LV (LVR), which accounts for a 5 ms refractory period, were also calculated as described previously (Shinomoto et al., 2003, 2009). The Fano factor was calculated by computing spike counts in 100 ms bins, then dividing the spike count variance by the mean spike count (Purcell et al., 2012b). Response field characteristics (maximum response, preferred location, and tuning width) were calculated by fitting a unimodal Gaussian function to the mean responses to the eight target locations. Response field characteristics were calculated separately for visual epochs (50–150 ms after target presentation) and movement epochs (50 ms preceding saccade initiation). Response field values were excluded from analysis if the Gaussian tuning function was unable to fit well (*r* ^2^ < 0.5).

### Cross-validation

To verify the accuracy of the consensus clustering algorithm, a leave-one-out classification procedure was used. A singular value decomposition classifier (SVD) classifier with a linear kernel was trained. To preserve the consensus metrics, the basis for the classifier training set was the composite pairwise distance matrix. However, this matrix was underspecified, so principle components of the pairwise distance matrix were calculated. The classifier was trained, for example, for the first principle component, the first and second components, and the first through third components for the first 100 principle components. The cumulative variance explained ranged from 43.4% to 93.4%. Due to the possible presence of groups with few members, this classifier was trained on all units but one, and the remaining unit was tested. No explicit regularization was performed when training the classifier. Only units that were assigned a category by the consensus clustering algorithm were included (*n* = 422 of 466, 90.6%).

## Results

This analysis is based on 466 units sampled in FEF from three macaque monkeys performing memory-guided saccades in pursuit of other research aims.

### Traditional response categorization

**Figure 2.**
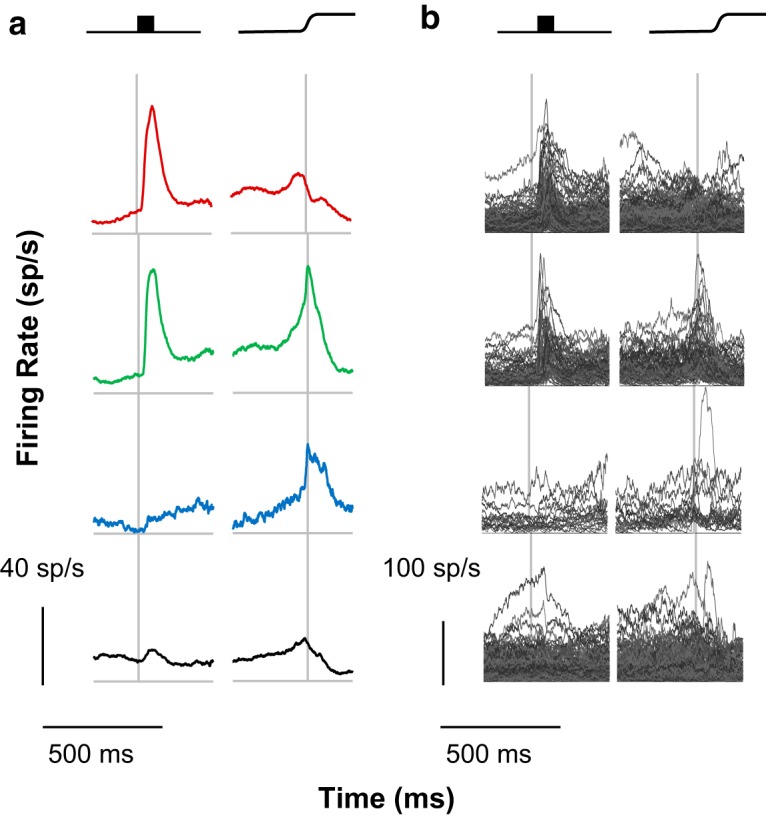
Traditional classification. The current sample was classified according to traditional criteria. ***a***, Group mean SDFs for visual, visuomovement, movement, and unclassified neurons are depicted from top to bottom, with left panels aligned on stimulus onset and right panels aligned on saccade. Here and in subsequent figures, the categories of neurons are arranged on a visual-to-motor axis, and colors are assigned such that red indicates visual activity and no movement activity, green indicates both visual and movement activity, and blue indicates movement activity without visual activity. Black indicates unclassified neurons. Scale bars for response magnitude and time are shown at the bottom left. ***b***, Individual spike density functions comprising each category. Scale bars for response magnitude and time are shown at the bottom left.

First, units were categorized based on traditional criteria (Bruce and Goldberg, 1985; Schall, 1991), and the canonical visual, movement, and visuomovement units were identified (Fig. 2). Of 466 neurons sampled, 210 (45.1% of sampled neurons, 70.2% of presaccadic modulated neurons) were identified as visual, 16 (5.4%, 5.4%) were movement related and 73 (15.7%, 24.4%) were visuomovement related. The remainder exhibited other patterns of modulation. Earlier studies using different tasks than we used here reported more diversity among the three major groups. We will consider this in the Discussion. For now, this simpler categorization facilitates the motivation of this approach.

While the average discharge rates of these categories were as expected, the SDF of the individual units categorized into each type exhibited considerable variation. For reference with subsequent analyses, the *RoV* value was 50.89. Thus, while the traditional categorization methods captured general trends in the modulation patterns of FEF neurons, additional variation was present but unaccounted for.

### Cluster pipeline 1: *z* score relative to baseline, mean measurement, Euclidean distance

**Figure 3.**
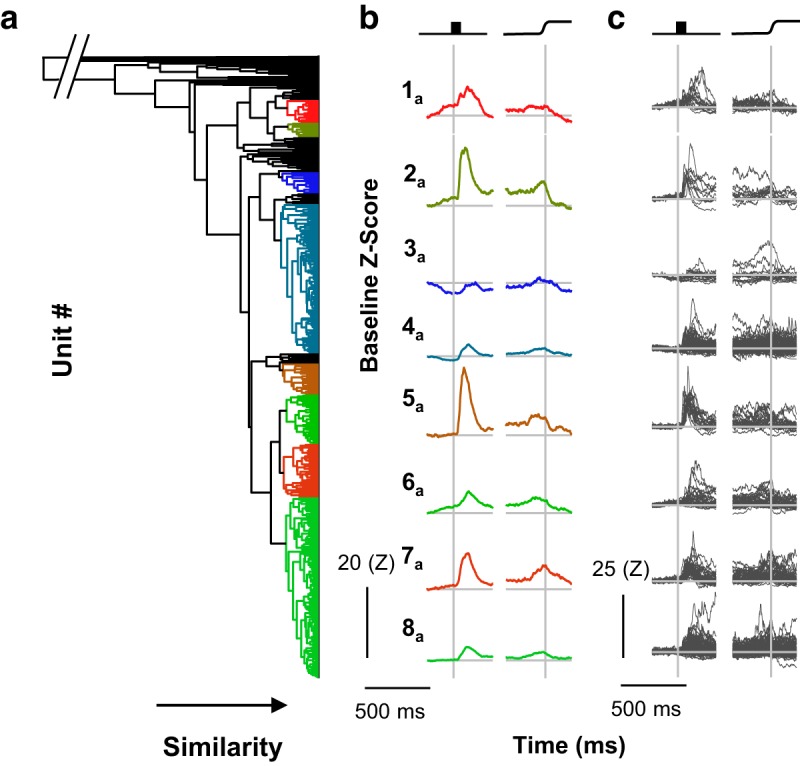
Cluster pipeline 1. Neurons were categorized via cluster analysis scaling by the *z* score relative to the baseline, mean measurement, and Euclidean distance. ***a***, The dendrogram resulting from cluster pipeline 1 shows the eight identified categories. Horizontal distance indicates pairwise similarity, with individual neurons on the right and full agglomeration on the left. Colors indicate categories and are arbitrarily assigned on a visual-to-motor axis as in Figure 3. The break at the top left indicates that the final agglomeration takes place at a point that prevents the visibility of categories. ***b***, Category means are plotted aligned on stimulus onset and saccade. Each category was given an arbitrary numerical identifier for convenience and are ordered according to their position in the dendrogram. Scale bars are shown at the lower left. ***c***, Individual neurons comprising each category aligned on stimulus and saccade. Scale bars are shown at the lower left.

To begin accounting for this excessive variability, a cluster analysis was performed on the SDFs that were *z* scored based on the mean and SD of the baseline firing rate. This captured the definition of visual and movement activity used above. However, the data-driven clustering procedure revealed functional categories that are similar in their firing rate modulations in more than just two epochs. Based on the mean firing rate in the six specific task epochs described in Materials and Methods, each unit was represented as a six-element vector. Based on Euclidean distance measures of pairwise distances, eight categories of units, numbered 1_a_ to 8_a_ to distinguish this set of results, were found (Fig. 3). Unlike the traditional categorization scheme, two categories of visual units were identified as categories 1_a_ and 4_a_ [17 of 466 (3.7%) and 11 of 466 (2.4%), respectively]. Both categories had modest visual responses and no perisaccadic activity. They were differentiated by the presence or absence of anticipatory activity before the target appeared and by delay period activity. An additional category, category 5_a_ [37 of 466 (7.9%)], had a robust visual response with weak presaccadic ramping. Four categories of units had both visual and presaccadic responses. Two of these categories had robust visual responses and intermediate presaccadic ramping: categories 2_a_ and 7_a_ [40 of 466 (5.2%) and 112 of 466 (24.0%) respectively]. These categories were differentiated by the return to baseline after the saccade: category 7_a_ had a typical slow return to baseline, whereas category 2_a_ returned to baseline almost immediately. The other two categories, 6_a_ and 8_a_ [135 of 466 (29.0%) and 16 of 466 (3.4%) respectively], had only modest firing rate modulation in both epochs and were distinguished by the presence (8_a_) or absence (6_a_) of anticipatory activity. The final category, 3_a_ [24 of 466 (5.2%)], did not show firing rate modulation during the trial. This demonstrates that additional diversity is present in FEF firing patterns that has been unaccounted for in the traditional scheme. However, this clustering approach failed to identify purely movement neurons or postsaccadic neurons.

The eight categories did not match the three traditional categories in FEF that should have been recovered through cluster analysis. However, similar to the traditional categorization scheme, these category means captured some general trends of the individual units comprising the categories. Variation was reduced (*RoV* = 18.36 relative to 50.89 for the traditional classification), but considerable variation was still evident. This was particularly pronounced in categories 4_a_ and 8_a_, which were also much larger categories than the other six. Thus, these categories seem to be “catch-all” categories. Other categories seem to be nearly identical, though are clearly seen as different groups in the dendrogram (e.g., categories 2_a_ and 5_a_). The dendrogram in Figure 3*a* also shows that units did not exhibit clear clustering. Instead, it appears as though small groups or individual units are progressively grouped together such that, at the clustering step when eight categories meet the membership criterion, 74 of 466 (16%) units were still uncategorized. That is, for the number of categories identified via the gap procedure of Tibshirani et al. (2001), 16% of neurons were so dissimilar to each other and the eight categories that they could not be placed in any of the eight categories or form a ninth separately. This may be so because the variation of the SDF of the identified units was high (mean skewness = 1.35). Together, these considerations indicate that this clustering procedure is insufficient.

### Cluster pipeline 2: nonscaled SDF, mean measurement, correlation distance

To account for more of the excessive variability, a cluster analysis using a correlation distance measurement was performed on the non-scaled data. This approach captures relative rather than absolute changes in firing rate. That is, if two units were similarly modulated but have different firing rates, this procedure treated them as members of the same category. In other words, this approach emphasized the pattern of modulation of FEF neurons rather than the absolute discharge rate.

**Figure 4.**
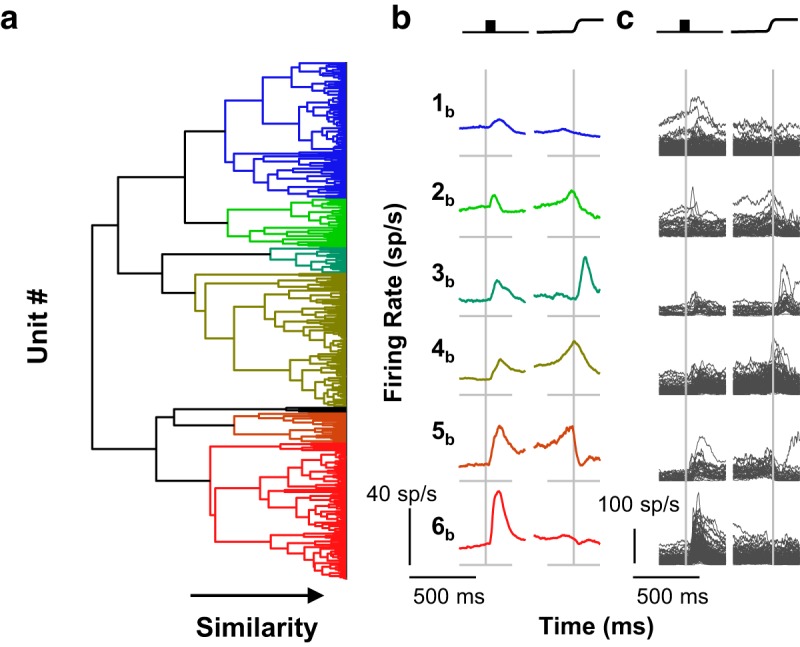
Cluster pipeline 2. Neurons were categorized via cluster pipeline using no scaling procedure, mean measurement, and correlation distance. Conventions for ***a*** through ***c*** are as in Figure 3.

This procedure identified six categories, 1_b_ to 6_b_ (Fig. 4). These categories did not match the three traditional categories; movement neurons are missing. Instead, each of the six identified categories demonstrated modulation following visual stimulation to different degrees. Two of these categories, 1_b_ (123 of 466) and 6_b_ (122 of 466), showed visual modulation only and were differentiated by the baseline firing rate and degree of visual modulation. Three categories had both visual and movement-related activity: 2_b_ (28 of 466), 4_b_ (44 of 466), and 5_b_ (24 of 466). Category 2_b_ had modest visual modulation and no delay activity; category 4_b_ had modest visual activity and some delay activity; and category 5_b_ had robust visual modulation, prominent delay activity, and presaccadic ramping, but its activity fell off dramatically at the time of the saccade. The final category, 3_b_ (121 of 466), had modest visual modulation and a sharp postsaccadic transient. Four of 466 neurons were placed in no category.

Considerable variability within categories remained and in fact increased relative to cluster pipeline 1 (*RoV* = 29.70 as opposed to 18.36), though the category means did capture category trends. This variability may be due to the skewed firing rates (mean skewness, 2.09), which allowed few units in one category to drive the modulations apparent in the category means. For example, the inclusion of some units with large visual responses and high firing rates in category 2_b_ was a driving factor in the modest visual activity seen in the category mean. Thus, both the *z* score relative to baseline and unscaled firing rates may be ineffective for comparing across units, although they are useful for assessing within-unit modulation. However, the appearance of the dendrogram for this clustering outcome should be noted (Fig. 4*a*). Unlike that from pipeline 1, a more sensible structure is apparent when this combination of clustering parameters was used; fewer neurons are nonclassified, and the categories visibly self-segregate.

### Cluster pipeline 3: *z* score relative to whole-trial, mean and slope measurement, and correlation distance

**Figure 5.**
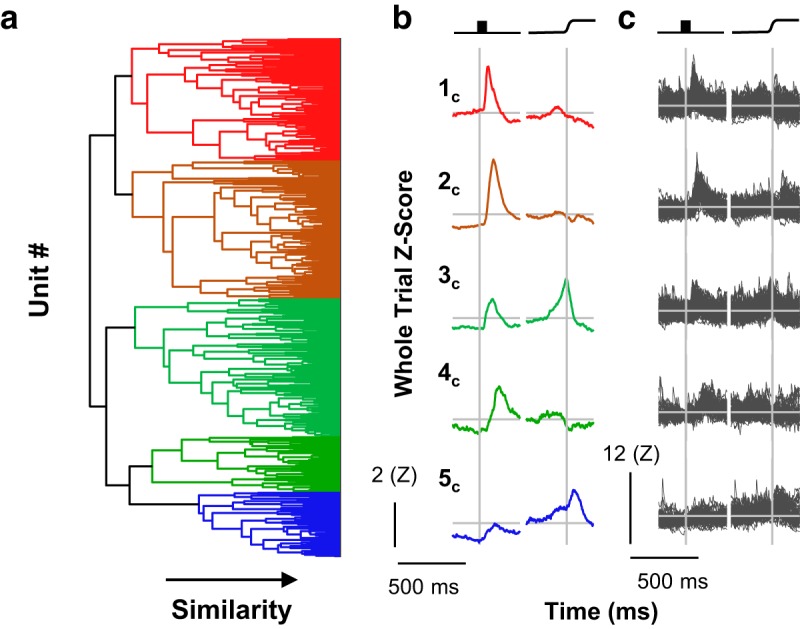
Cluster pipeline 3. Neurons were categorized via cluster analysis scaling by the *z* score relative to the whole trial, mean and slope measurement, and correlation distance. Conventions for ***a*** through ***c*** as in Figure 3.

To account for more of the variability in modulation patterns, a different scaling procedure was used: *z* scoring across the entire trial and measuring the SDF with both the means of the SDF and the slopes during the relevant epochs were considered. The agglomerative clustering algorithm identified five categories, 1_c_ to 5_c_ (Fig. 5). Three of these categories had visual activity only: 1_c_ (110 of 466), 2_c_ (124 of 466), and 4_c_ (124 of 466). Category 4_c_ had pronounced delay activity. The other two were distinguished by the time of peak visual response, with the visual activity of category 1_c_ peaking earlier, and that of category 2_c_, later. Category 3_c_ (50 of 466) had robust visual and presaccadic modulation but did not have delay activity. The final category, 5_c_ (58 of 466), showed robust presaccadic ramping and only modest, if any, visual activity. It should be noted that the presaccadic ramping activity in category 5_c_ peaked after the saccade and showed a slow reduction of firing rate back to baseline, whereas the category with both visual and saccadic responses had a peak perisaccadic activity at the time of the saccade followed by a sharp return to baseline, but this sharp return is not as pronounced as the “clipped” movement neurons in category 5_b_. This indicates that the additional diversity evident in visual inspection of discharge rate modulation patterns is tangible and identifiable. Further, of the three cluster pipelines, pipeline 3 produced the classification most similar to the traditional. Visual and visuomovement categories were identified as well as a putative pure movement group (category 5_c_).

The range of values through this scaling was smaller and is less skewed (mean skewness, 0.73), suggesting that this scaling method provided a more equitable cross-unit comparison. As with pipeline 2, these categories are apparent in the dendrogram structure, though some additional heterogeneity can be observed, particularly among categories 1_c_ and 2_c_. This may explain, in their particular cases, the seeming similarity between the two; it could be that splitting further would reveal additional heterogeneity, and, in the case of the category 3_c_, further splitting could reveal a second visuomovement group without clipped activity as well as a pure movement category. However, it should be noted that. unlike cluster pipelines 1 and 2, the category means were much more representative of the members (*RoV* = 5.19 as opposed to 18.36 and 29.70 for cluster pipelines 1 and 2, respectively).

### Consensus clustering

**Figure 6.**
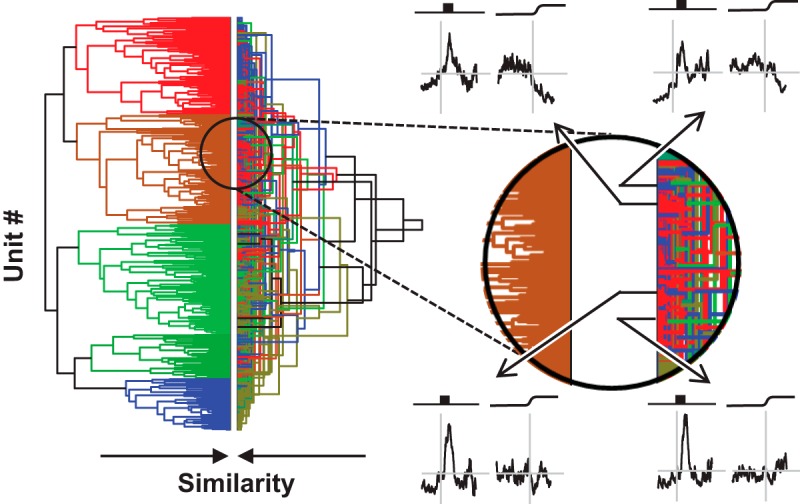
Comparison of analysis pipelines. Left: Dendrograms from cluster pipelines 2 (right) and 3 (left). They are shown side-by-side to highlight the similarities and differences in the respect categories. The dendrogram for cluster pipeline 3 is identical to the dendrogram in this figure. The dendrogram for cluster pipeline 2 shows the same results as in Figure 5; however, the vertical arrangement was reordered so that common units are horizontally aligned in both dendrograms. Where common colors are horizontally aligned, units were assigned to the same category. Where different colors are horizontally aligned, units were assigned to different categories. Although horizontal alignment of some dendrogram elements is evident, the disagreement between the two dendrograms is more prominent. The extent and nature of this disagreement is illustrated in the expanded view of the dendrogram on the right. SDFs of four representative units are shown. Through analysis of pipeline 3, all four units were placed in category 2c, which characterized by a pronounced visual response and weak perisaccadic suppression (left dendrogram). Through analysis of pipeline 2, three of the units were placed in category 6b, which is characterized by a pronounced visual response and weak perisaccadic suppression (red, right dendrogram), whereas the unit shown at the upper right was placed in category 1b, which is characterized by a weak visual response and no perisaccadic modulation (blue, right dendrogram). Thus, the two analysis pipelines provide overlapping, but far from identical, categorizations. Which categorization is correct is uncertain.

We now address the problem of individual units being members of different categories following different analysis paths (Fig. 6). This occurs because different preprocessing pipelines resulted in different distance matrices on which the agglomerative clustering algorithm operates. Consequently, a given pair of units could be members of the same category following one pipeline but members of different categories following another pipeline. For example, all four individual units shown in Figure 6 belong to category 2_c_, but only three belong to category 6_b_; the unit on the top right belongs instead to category 1_b_. With no cluster pipeline being more confidently motivated or more certainly correct than another, should all four units be considered members of the same category or not? Nevertheless, assuming the existence of ground-truth categories, consistent with anatomic constraints, units that are actually members of the same ground-truth category should have small pairwise distances regardless of a scaling or clustering procedure. Likewise, units that are members of different ground-truth categories would have small pairwise distances only as an artifact of particular measurement parameters and clustering algorithm.

To address this fundamental problem, we used a second-order clustering procedure known as consensus clustering (Strehl and Ghosh, 2002; Goder and Filkov, 2008). We created a composite distance matrix by *z* scoring individual distance matrices from all of the preprocessing pipelines (Fig. 1*f*) and then calculated the median distance across all preprocessing pipelines. This composite distance metric was used to identify units that were consistently similar to one another across preprocessing pipelines and clustering algorithms. The agglomerative clustering algorithm was applied to this distance matrix to identify robust categories of units superordinate to any individual cluster analysis. Clusters were continually split until either of two membership criteria were no longer satisfied: (1) minimum number of units per cluster; and (2) maximum proportion of unclustered units.

**Figure 7.**
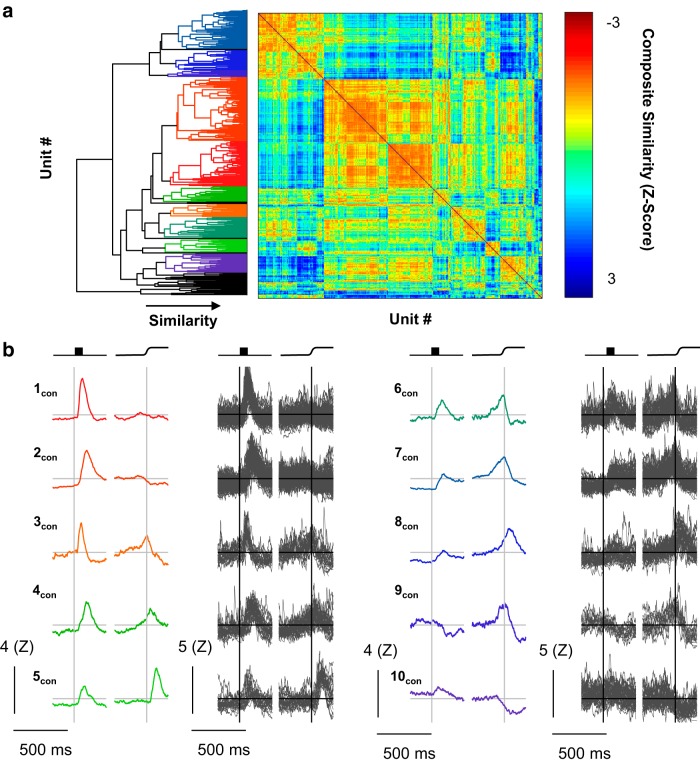
Consensus clusters. Consensus clusters were identified by creating a composite distance matrix and applying the agglomerative clustering procedure to this matrix. ***a***, The resulting dendrogram is shown abutting the composite distance matrix. Colors in the dendrogram are assigned as in Figures 3–6. Color in the distance matrix is indicative of composite similarity. Warm colors (low composite *z* score) indicate consistently similar units, whereas cool colors indicate consistently different units (high composite *z* score). ***b***, The category mean SDFs (columns 1 and 3) and the individual SDFs (columns 2 and 4) comprising them are shown aligned on stimulus onset (left) and saccade (right). Scale bars are shown at the lower left of each column. Arbitrary category labels were assigned for convenience.

Consensus clustering identified 10 categories, clearly distinguished in the dendrogram and evident in the distance matrix (Fig. 7). Of 466 neurons, 43 (9.2%) were not placed in any category. These categories were robust and consistent (*RoV*, 3.91). Even with the penalty for overclustering in the *RoV* metric, consensus clusters account for more of the variability in the neural data than the classification produced by the best individual classification.

#### Categories with visual responses only

We identified two categories of neurons that had visual, but not saccade-related, activity. These categories, 1_con_ (74 of 466) and 2_con_ (105 of 466), showed flat baseline activity and a sharp visual transient. The time of peak firing rate differentiated these two categories, with mean peak latencies of 74 ms (1_con_) and 136 ms (2_con_). Also, category 2_con_ had persistent delay activity until the saccade.

#### Categories with visual and saccade-related facilitation

We identified five categories of neurons with both visual and presaccadic increases in firing rate. Two of these categories, 3_con_ (21 of 466) and 4_con_ (25 of 466), showed marked increases in firing rates following visual stimulation and were distinguished by the time of peak visual activity (mean values of 70 and 161 ms, respectively). They were also distinguished by the time and character of the presaccadic ramping. The firing rate of category 3_con_ neurons peaked at the time of the saccade and quickly returned to baseline, whereas the firing rate of category 4_con_ peaked after the saccade and returned to baseline more slowly. Two of the three remaining categories, 6_con_ (35/466) and 7_con_ (64/466), also had clipped movement activity with late, weak visual responses. These two categories are differentiated by the absence (6_con_) or presence (7_con_) of delay period activity. The final category, having both visual and movement activity, 8_con_ (33 of 466), showed only modest visual activity and may be more accurately described as a pure movement category. In either case, the movement-related activity peaked just after the saccade but returned slowly to baseline. An additional category, 5_con_ (23 of 466), was not movement related per se, but exhibited a strong postsaccadic transient with a modest, early visual response.

#### Categories with response attenuation

Two categories of units showed distinct decreases in firing rate. The first of these, 9_con_ (11 of 466), was unique in having an “off” response to visual stimulation, but it also showed clipped presaccadic ramping that peaked just before the saccade. This provides a fruitful contrast with the final visuomovement category, category 8_con_, which showed only a modest increase in firing rate but robust, unclipped perisaccadic ramping. This distinction may be a useful criterion in future studies examining the differences in stimulus-driven or goal-directed saccades. The second category with an off response, 10_con_ (31 of 466), showed little or no visual modulation, but was sharply inhibited around the time of the saccade, which is characteristic of fixation neurons.

#### Relation to other functional characteristics

To preclude that these categories are accidental and arbitrary, we quantified several other characteristics of each neuron. These included typical measures such as response field size and center location, baseline discharge rate, and maximal response for both the visual and motor periods of modulation. These were supplemented by the following discharge variability metrics: Fano factor, CV, CV_2_, LV, and LVR accounting for a 5 ms refractory period. Finally, spike width was also measured.

First, omnibus Kruskal-Wallis tests were performed for each of these factors. Factors with significant differences include visual response field width (χ^2^_(9274)_ = 18.300; *p* = 0.032), maximum visual response (χ^2^_(9267)_ = 20.881; *p* = 0.013), and baseline firing rate (χ^2^_(9401)_ = 28.600; *p* = 0.001).

Second, to take a more targeted approach, categories 1_con_ and 2_con_ were considered visual; categories 3_con_, 4_con_, 6_con_, and 7_con_ were considered visuomovement; and categories 8_con_ and 9_con_ were considered movement related. The analyses were repeated separately for these sets of consensus categories. Among the visual categories, receptive field width was significantly larger for category 2_con_ (60.6 ± 31°) than for category 1_con_ (52.4 ± 34.8°; χ^2^_(1136)_ = 4.568; *p* = 0.033). For movement-related categories, category 8_con_ had significantly wider spikes (266.0 ± 122.5 μs) than category 9_con_ (173.6 μs ± 67.2 μs; χ^2^_(1,42)_ = 5.625; *p* = 0.018). No significant differences or trends were identified among the visuomotor categories. Thus, these consensus clusters identify differences in neuron types, even when the factors for which differences were identified were not included as parameters for clustering.

### Cross-validation analysis

Clustering analyses can be problematic because the algorithms involved will yield as many categories as are requested from any data sample, regardless of the underlying category structure. The use of a minimum membership criterion and a maximum uncategorized percentage criterion aim to mitigate this, but the contribution of these criteria to eliminating the problem of oversplitting the categories cannot be directly quantified. Instead, to quantitatively assess the quality of the categorization, a classification analysis was used. This analysis uses a leave-one-out cross-validation approach in which a classifier is trained on the consensus category membership of all recorded units except one, then assigns that remaining unit to one of the consensus categories. To prevent underspecifying the classifier, principle components of the composite consensus matrix were used (see Materials and Methods). Classification accuracy is assessed by the percentage of units that, when left out of the training set, are assigned to the same category as that specified by the full consensus clustering algorithm.

**Figure 8.**
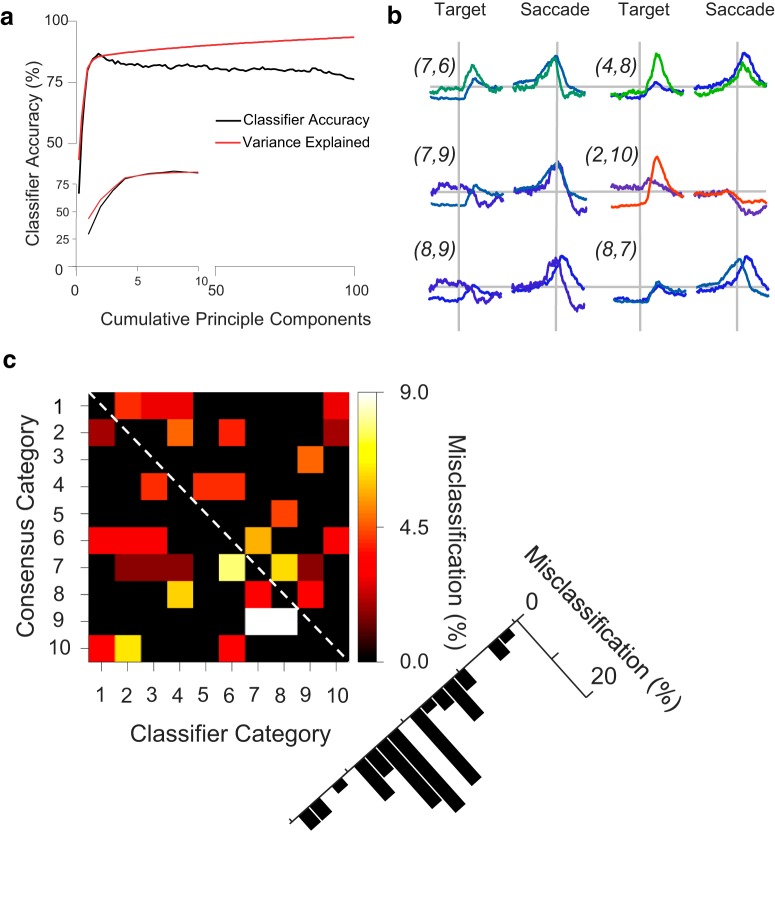
Cross-validation analysis. Leave-one-out cross-validation was performed separately for 1 through 100 principle components of the composite distance matrix. A singular value decomposition classifier (SVD) classifier with a linear kernel was trained on the principle components of all but one neuron, and that remaining neuron was categorized. ***a***, Classifier accuracy as a function of cumulative principle components, plotted up to 100 principle components (main plot) and for first 10 (inset). Peak accuracy (86.7%; chance accuracy was 10%) was achieved using eight principle components, which corresponds to a plateau of variance explained by additional components. ***b***, Superimposed mean SDFs for pairs of categories that were most frequently misclassified, labeled as (classifier category, consensus category). Although the category SDFs clearly differ, the source of misclassification is apparent through particular common features between pairs. ***c***, Matrix showing the incidence and nature of misclassifications. Matrix rows distinguish the consensus algorithm categories; numbers correspond to consensus cluster spike density functions. Matrix columns distinguish the classifier categories. If the classifier were perfectly accurate, then the matrix would be entirely black, indicating no misclassification. The black cells along the unity diagonal (classifier column C = consensus category row R, indicated by dashed line) are the 86.7% of neurons for which the classifier correctly identified the consensus algorithm category; they are not misclassified. Black cells off of the unity diagonal (C ≠ R) indicate that the classifier did not misclassify neurons in row R as belonging to column C. Colored cells off of the unity diagonal indicate that the classifier misclassified neurons in row R as belonging to column C. The color map shows percentages of misclassified neurons relative to the count of consensus category R. Misclassified neurons can be identified, for example, as an adjacent category (C = R + 1) or two categories away (C = R + 2). The percentage of total misclassifications that were assigned to C = R + *n* are shown to the lower right. Misclassifications are most common for adjacent categories (C = R ± 1) and are generally progressively less common with greater category separation.

Peak accuracy was 86.7%, which was achieved when the classifier was trained with eight principle components, after which cumulative variance explained remained on a plateau (Fig. 8*a*). The units misclassified by the classifier were identified. The percentage of units from each consensus category misclassified in each classifier category is depicted as a matrix of consensus category rows and classifier prediction columns (Fig. 8*c*). Generally, misclassified units were found in adjacent categories, with misclassifications becoming less frequent as the categories become further apart (Fig. 8*c*). Given the purposeful ordering of these units on a visual–motor spectrum, this is not surprising. The highest percentage of misclassified units (9.1%) was in category 9_con_, which also has the fewest members according to the consensus clustering algorithm.

Category pairs with frequent misclassifications are superimposed in Figure 8*b*. Categories 6_con_ and 7_con_ both show robust movement activity and similar visual activity. Categories 4_con_ and 8_con_ have nearly identical late-peaking movement activity. Categories 7_con_ and 9_con_ also have highly similar movement related activity. Categories 2_con_ and 10_con_ both have suppressed activity at the time of saccade. Categories 8_con_ and 9_con_ are the two categories of nearly pure movement activity. Finally, categories 7_con_ and 8_con_ both have weak visual activity as well as robust movement activity, although with different timing. Overall, though there are differences among these pairs of categories, there are features that explain why misclassifications could be made between these pairs.

To confirm that the peak classification accuracy was indeed greater than the nominal chance value of 10%, the above procedure was used with the category assignments randomly shuffled. For these shuffled assignments, the first eight principle components were used to train the classifier because this corresponds to the peak in classifier accuracy. Including additional components decreases accuracy, most likely due to overfitting. Shuffling was performed 1000 times. No randomized classification accuracy exceeded the empirical classification accuracy (mean = 8.2%; SD = 3.7%; range = 1.0–21.6%). This indicates that the consensus clustering algorithm categorizes neurons in a highly internally consistent manner. The robust reclassification of the original set also allows for new data to be categorized according to the present consensus categories.

## Discussion

We applied a consensus clustering technique and identified 10 robust functional categories in FEF based on modulation of discharge rates alone. This categorization includes but exceeds the traditional categories. We will discuss the relationship of the new functional categories to the traditional categorization and possible functional and anatomic implications of these consensus clusters. We will conclude by considering the limitations and extensions of these consensus clustering techniques.

### Correspondence with traditional functional categories

**Figure 9.**
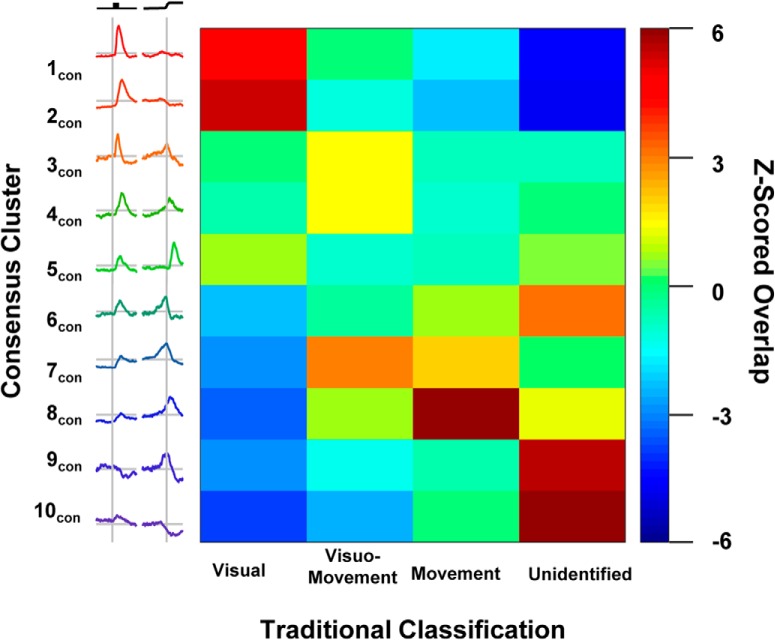
Relation to traditional classification. The consensus cluster assignments were compared with traditional classifications. The consensus clusters are depicted on the vertical axis, and traditional classification is depicted on the horizontal axis. The color in the heat map indicates the prevalence of neurons being classified in a given combination. For a given cell in the matrix, a warm color indicates that more neurons were assigned to both that consensus cluster and that traditional classification than expected by chance, green indicates that the expected number of neurons were assigned to both categories, and a cool color indicates that fewer than expected neurons were assigned to both categories. Cluster 1_con_ and 2 _con_ neurons were more often identified as visual cells and were rarely uncategorized. Cluster 3 _con_, 4 _con_, and 7 _con_ neurons were often identified as visuomovement cells. Cluster 8 _con_ neurons were more often identified as movement cells and not visual cells. Cluster 9 _con_ and 10 _con_ neurons were generally not categorized, but when they were they were not classified as visual cells.

To compare the traditional and this new categorization, we assessed their overlap by calculating the proportion of consensus clusters identified as visual, movement, visuomovement, or unclassified. The traditional scheme and our new consensus clustering procedure show significant overlap (Fig. 9; *ARI* = 0.0931, *p* < 0.001). Thus, our procedure complements the traditional categorization. In fact, although not sought specifically, both postsaccadic and fixation neurons were identified via consensus clustering. This unsupervised discovery increases confidence that consensus clustering identifies natural neural categories.

We now compare the proportions of different categories to previous surveys. Bruce and Goldberg (1985) identified 40% of their sample as visually related, 40% visuomovement, and 20% movement only, not including postsaccadic and fixation neurons. Postsaccadic and fixation neurons account for 17% and 7% of their total sample, respectively. Schall (1991) identified 17% of the sample as visual, 41% as visuomovement, 22% movement, and 13% postsaccadic. When using the traditional categorization, 45% of the current sample was visual, 16% was visuomovement, and only 3% was movement. This proportion of visual neurons with respect to the whole sample is consistent with the findings of Bruce and Goldberg (1985), but not with Schall (1991). The proportions of neurons are more similar to earlier descriptions when consensus categories are considered with 38% visual, 31% visuomovement, 9% movement, 7% fixation, and 5% postsaccadic. Still, visuomovement, movement, and postsaccadic neurons are still underrepresented in the current studies relative to previous studies. This under-representation of movement-related neurons seems curious, for these are thought to be large pyramidal neurons in layer 5 (Fries, 1984; Segraves and Goldberg, 1987; Sommer and Wurtz, 2001), which should be easier to isolate. Similarly, fixation neurons are also layer 5 pyramidal cells (Izawa and Suzuki, 2014), but are instead found in the same proportion as in the study by Bruce and Goldberg (1985). Perhaps the linear electrode array failed to sample layer 5 neurons. Future reconstructions of the recording sites will determine whether laminar differences can explain the differences in proportions of neurons.

Another possible explanation for differences in category proportions concerns the nature of the electrodes. The neural spiking analyzed for this study was obtained with linear electrode arrays (Plexon U-probe and Neuronexus vector probe). The studies cited above sampled neurons with a variety of sharp electrodes including glass-coated, platinum-iridium, and tungsten. The differential sampling characteristics of various electrodes in FEF require further investigation.

A third possibility involves the eccentricity represented by the neuron samples across studies. The central visual field is represented laterally and peripheral field medially in FEF and rostrally adjacent cortex (Suzuki and Azuma, 1983), and RF size increases with eccentricity (Mayo et al., 2015). Lateral and medial FEF have qualitatively and quantitatively different patterns of connectivity (Schall et al., 1995a; Babapoor-Farrokhran et al., 2013; Markov et al., 2014). Convergence from the dorsal and ventral processing streams occurs in lateral but not in medial FEF. Lateral FEF, which is responsible for generating short saccades, receives visual afferents from the foveal representation in retinotopically organized areas, from areas that represent central vision in inferotemporal cortex and from other areas having no retinotopic order. In contrast, medial FEF, which is responsible for generating longer saccades, is innervated by the peripheral representation of retinotopically organized areas, from areas that emphasize peripheral vision or are multimodal and from other areas that have no retinotopic order or are auditory. Hence, neural spiking samples from lateral and medial FEF are likely to differ in a variety of as yet uncertain ways. Here, all stimuli were placed at 8° eccentricity, whereas tested locations in the study by Bruce and Goldberg (1985) ranged from 5° and 45°, and the study by Schall (1991) used 15° horizontal and 8° vertical. Systematic mapping across eccentricities is needed to resolve this question.

A fourth possibility involves the nature of tasks and reward contingencies. As noted above, the particular memory-guided saccade task used here is not identical to tasks used in previous studies. Factors like stimulus luminance, chromaticity, and contrast will need to be explored systematically (Krock and Moore, 2016). Moreover, FEF neurons are sensitive to reward contingency (Roesch and Olson, 2003) and other cognitive processes (Ferrera et al., 2009; Middlebrooks and Sommer, 2012; Teichert et al., 2014).

Functional differences can arise from structural differences in connectivity (Schall et al., 1995a; Markov et al., 2014), in morphology (Lewis and Lund, 1993), and in biophysical properties (McCormick et al., 1985; Connors and Gutnick, 1990; Krimer et al., 2005 Vigneswaran et al.,^2011^; Casale et al., 2015; Rocco et al., 2016), although the relative contributions of these factors are unknown in FEF. Indeed, using a related consensus clustering approach on physiologic measures from monkey prefrontal cortex, Ardid et al. (2015) reported four broad spiking, putative pyramidal cell classes and three narrow spiking, putative inhibitory cell classes, which were distinguished by sparse, bursting, or regular spike trains. Using a related agglomerative clustering approach, Zaitsev et al. (2012) also reported four classes of neurons visually identified as pyramidal cells *in vitro* that were also distinguished by sparse, bursting, or regular spike trains. These authors also identified three classes of inhibitory interneurons identified morphologically *in vitro* that were distinguished by firing rate variability measures, and these classes were each associated with calcium binding proteins parvalbumin, calbindin, or calretinin.

In this sample, we found no significant differences in measures of firing rate variability across the consensus categories. However, spike width differed between the two movement-related categories, indicating some differences in biophysical characteristics. Curiously, spike width did not differ between consensus clusters identified as visual, movement related, and visuomovement, which is at odds with findings from previous studies (Cohen et al., 2009a; Ding and Gold, 2012, Thiele et al., 2016). Further investigation is necessary to determine the reason for this difference. To that end, the consensus clustering method can be extended to incorporate biophysical characteristics such as spike polarity and phase (Gold et al., 2009), spike width (Cohen et al., 2009a; Vigneswaran et al., 2011), spike timing patterns (Holt et al., 1996; Nawrot et al., 2008; Cohen et al., 2009a; Shinomoto et al., 2009), and Fano factor (Purcell et al., 2012b).

### Possible functional implications

Most of the consensus categories were characterized by pronounced perisaccadic activity. Many such neurons also had pronounced visual responses (3_con_, 4_con_, 5_con_, 6_con_, and 7_con_) and will be discussed below. Categories 8_con_ and 9_con_ were distinguished by (1) weaker modulation of opposite signs after the target appeared, (2) time of peak saccade-related activity, (3) duration of activity after the saccade, and (4) spike width. Both patterns of modulation have been reported previously in FEF (Bruce and Goldberg, 1985; Hanes and Schall, 1996; Everling and Munoz, 2000; Sommer and Wurtz, 2001; Lawrence et al., 2005). The peak activity of category 9_con_ neurons coincides with saccade initiation, and discharge rate is reset by saccade termination. Such clipped neurons have been reported in FEF by one study (Hanes et al., 1995) but not another (Segraves and Park, 1993). Clipped movement neurons have been associated with saccade dynamics in superior colliculus (Waitzman et al., 1991). Confirming the presence of clipped movement neurons in FEF would substantiate the hypothesis that FEF contributes to the dynamics of saccade production (Schiller et al., 1987; Dias and Segraves, 1999; Peel et al., 2014). The activity of category 8_con_ has a more sluggish relationship to saccade timing, peaking after the saccade and resetting well after saccade termination. These properties are inconsistent with a direct role in saccade production. Further investigation with other task conditions is necessary. For example, another approach to determining whether individual neurons are involved in controlling saccade initiation involves testing with the saccade-countermanding task (Hanes et al., 1998; Murthy et al., 2009). Alternatively, distinct functions may be revealed when planning saccade sequences (Phillips and Segraves, 2010).

Relative to category 8_con_, category 9_con_ neurons had significantly narrower spike widths. This does not entail, necessarily, that 9_con_ neurons are inhibitory interneurons. In primary motor cortex, some identified corticospinal pyramidal neurons have narrow spike widths (Vigneswaran et al., 2011) presumably because these neurons have a fast potassium channel K_v_3.1b subunit (Ichinohe et al., 2004). If corticotectal and corticopontine neurons are analogous to corticospinal neurons, then spike width may be misleading in the identification of projection neurons. However, whether layer 5 neurons in FEF stain positively for fast potassium channels is unknown.

The analysis also identified a consensus cluster with characteristics of fixation neurons (10_con_). These neurons seem involved in the active maintenance of fixation and may release inhibition on presaccadic movement neurons to produce saccades (Segraves and Goldberg, 1987; Hanes et al., 1998). The cluster exhibited a modest visual response. If these neurons do indeed inhibit presaccadic movement neurons, then this could be the origin of the brief reduction of discharge rate characteristic of the movement neuron consensus cluster 9_con_. Greater diversity of this category may be found if tested with pursuit eye movements (Izawa and Suzuki, 2014).

Most of the consensus categories were characterized by pronounced visual responses. This is consistent with previous descriptions of FEF neural properties (Mohler et al., 1973; Bruce and Goldberg, 1985; Schall, 1991). Two consensus clusters were distinguished by strong visual responses and no modulation associated with saccades (1_con_ and 2_con_). Category 1_con_ had an earlier peak response and no delay activity, whereas category 2_con_ had a later peak response and clear activity during the memory delay. The receptive fields of category 1_con_ were narrower than those of category 2_con_. The diversity of FEF visual responses along multiple dimensions is well known in the early visual pathway. For example, the well known distinction of transient and phasic visual responses (Cleland et al., 1971) is evident in FEF (Sato et al., 2001). The diversity of visual responses likely arises principally from the diversity of connectivity. As noted above, FEF is reciprocally connected with an unusually large number of extrastriate visual cortical areas, and pronounced differences in connectivity distinguish medial and lateral FEF (Schall et al., 1995a; Babapoor-Farrokhran et al., 2013; Markov et al., 2014). Subcortical afferents can also influence visual responses; neurons in FEF that are activated orthodromically by SC stimulation have visual and saccade-related responses (Sommer and Wurtz, 1998). The extent to which visual response properties vary with cortical and subcortical connectivity is unresolved. The diversity of visual responses in FEF also relates to the variety of cortical areas in which FEF axons terminate. For example, V4 is influenced by visual neurons in layer 2/3 of FEF (Pouget et al., 2009; Noudoost and Moore, 2011; Gregoriou et al., 2012). However, FEF neurons projecting to V4 receive input from area 46, whereas the FEF neurons projecting to MT receive input from area 46 plus supplementary eye field. (Ninomiya et al., 2012). Thus, intracortical projections from FEF convey different signals. How much such signals vary across cortical targets of FEF efferents is unknown.

Most of the categories with visual responses were also characterized by modulation associated with saccade production. These are typically referred to as visuomovement neurons. While these data support no conclusions about the unique functional contributions of the four visuomovement categories, several characteristics warrant discussion. First, categories 3_con_ and 4_con_ have noticeably stronger visual responses than categories 6_con_ and 7_con_. Perhaps categories 3_con_ and 4_con_ occupy an earlier position in the visuomotor transformation. The saccade-related activity of these two categories is similar, so they may contribute equally to the production of saccades. However, the timing of the peak visual responses differs in a manner similar to categories 1_con_ and 2_con_. The earlier peak activity of category 3_con_ is consistent with receiving magnocellular pathway inputs, perhaps via category 1_con_ neurons. Meanwhile, the later peak activity of category 4_con_ is consistent with receiving parvocellular pathway inputs directly or via category 2_con_ neurons.

Categories 6_con_ and 7_con_ have similar visual responses but are distinguished by the magnitude of delay activity and the return to baseline following saccade. Category 6_con_ activity returns to baseline quickly after the saccade, indicating that this category may be more intimately involved in saccade dynamics than category 7_con_. This is consistent with the higher delay activity in category 7_con_, which may indicate that category 7_con_ is primarily involved in maintaining the stimulus location in working memory and therefore occupies an executive role as opposed to a direct role in saccade production. Of course, the lack of corroborating differences in other factors, including tuning characteristics, spike timing, and spike widths, may indicate that these categories are an excessive parsing of one continuum or that the measures are insensitive, but additional work is warranted to determine whether this is the case. For example, during visual search tasks, all visually responsive neurons respond equivalently to a target or a distractor in the receptive field (Schall et al., 1995b). Visual neurons with transient responses do not contribute to the selection of the target from distractors, but visually responsive neurons with prolonged activity do select the target of a search array when saccades are accurate (14% transient and 86% sustained; Thompson et al., 1996). When arbitrary stimulus–response mapping is required after visual search, many visually responsive neurons select the attended stimuli, while others select only the endpoint of the saccade (Sato and Schall, 2003). Further research is needed with this task and these categorization methods to determine how this previously observed distinction maps onto these new functional categories. When monkeys perform visual search for a target among distractors of fixed features, ∼50% of visually responsive neurons in FEF exhibit features selective from the initial response (Bichot et al., 1996). The same distinction may also explain feature-based attention differences identified in FEF neurons in other studies (Bichot and Schall, 2002; Gregoriou et al., 2009; Zhou and Desimone, 2011). Other distinctions among FEF neuron categories have been described in perceptual choice (Ding and Gold, 2012). Finally, the relationship of the dynamics of visuomovement and movement neurons to saccade initiation can be distinguished using the saccade-countermanding task (Ray et al., 2009).

Further insight may be gained by testing how the different categories contribute to eye–head coordination and visual–motor reference frame updating (Sajad et al., 2015, 2016). FEF neurons have also been implicated in remapping and trans-saccadic stability (Umeno and Goldberg, 1997; Crapse and Sommer, 2008, 2012; Shin and Sommer, 2012). These operations require information about the just executed saccade. A single consensus cluster of postsaccadic neurons was identified (5_con_). These neurons also had visual responses. Previous research has suggested that this type of neuron can support remapping and trans-saccadic stability by signaling the vector of the saccade that was just executed (Goldberg and Bruce, 1990). To produce sequences of saccades without visual guidance, the vector of the most recent saccade would be subtracted from the vector from the initial fixation point to the location of the second stimulus to account for the location of the second stimulus relative to the new fixation point rather than the point from which the location was initially encoded.

Antidromic stimulation studies agree that movement and fixation neurons project to the SC and brainstem, but they disagree about the projection of visual and visuomovement neurons (Segraves and Goldberg, 1987; Sommer and Wurtz, 2001). Perhaps the disagreement may be resolved by considering more refined categories of neurons. For example, perhaps only visuomovement neurons belonging to categories 6_con_ or 7_con_ with modest visual response, relative to categories 3_con_ and 4_con_, project from FEF to SC.

### Limitations and extensions of clustering procedures

Each of the individual clustering procedures is limited by the distance measure used to calculate pairwise similarity, by the measurement of the responses, and by the quality of the discharge rate samples. Different distance measures emphasize different aspects of the variability across units. Although Euclidean distance emphasizes similarity in absolute discharge rates, correlation distance emphasizes similarity in the pattern of modulation of discharge rates. Different measurements of the variation of discharge rate emphasize different aspects of the variability across units. Measuring the mean firing rate in different epochs captures absolute discharge rates but ignores dynamics. Measuring the slopes of the SDF in different epochs ignores absolute discharge rates. However, measuring both the means and slopes across many epochs or, indeed, using the SDF from the entire trial can expose the clustering algorithm to excessive incidental variation.

Different approaches to scaling the SDF across units emphasize different aspects of the variability across units. As shown in Figures 3–5, different methods of scaling the SDF across units can result in category means that do not accurately represent the individuals comprising those categories. Naturally, different scaling procedures emphasize useful information about the units. For example, *z* scoring the SDF based on the prestimulus baseline activity emphasizes the magnitude of modulation relative to the variation in the baseline. On the other hand, *z* scoring the SDF based on the entire trial reduces the skewed variation of discharge rates. Analytical choices must be made; hence, confidence in the outcome of every particular clustering pipeline can be questioned.

Consensus clustering increases confidence in distinctions identified across measurements and clustering procedures by minimizing spurious classifications arising from incidental analysis choices or unreliable data. Moreover, the consensus clustering approach affords the opportunity to include as many other measures and clustering procedures as desired. In particular, biophysical spiking properties are certainly useful for categorizing neurons. The eventual inclusion of such features will surely add complexity but should certainly approach an accurate account of the true diversity of functional neuron categories in FEF. A correct account of such diversity is necessary to support the next generation of microcircuit models (Mitchell and Zipser, 2003; Brown et al., 2004; Hamker, 2006; Heinzle et al., 2007).

## Synthesis

Reviewing Editor: Tatiana Pasternak, University of Rochester Decisions are customarily a result of the Reviewing Editor and the peer reviewers coming together and discussing their recommendations until a consensus is reached. When revisions are invited, a fact-based synthesis statement explaining their decision and outlining what is needed to prepare a revision will be listed below. The following reviewer(s) agreed to reveal their identity: Tirin Moore. Note: If this manuscript was transferred from JNeurosci and a decision was made to accept the manuscript without peer review, a brief statement to this effect will instead be what is listed below.

The manuscript revised in response to the JN reviews has now been reviewed by two reviewers. While both reviewers appreciated the need for a more principled classification scheme of FEF neurons, they raised reservations about the lack of explicit quantitative analyses to validate the clustering approach, the reservations also raised in the initial review for JN. These issues must be addressed before the manuscript can be considered for publication in eNeuro. These are outlined below:

1) Address the issue of single neuron isolation outlined by Reviewer 2 by providing additional proof that the neurons included in the classification were well isolated

2) Validate your cluster classification method with additional methods, such as classification analysis (linear or non-linear classifiers that use cross validation)

3) Following the suggestion of the initial JN Reviewer 2 who commented that the paper is “very dense”, please expand Fig. 1 to highlight the difference between the different methods.

REVIEWER 1

1) The authors should have additional proof of single neuron isolation

2) They should consider some methods such as classification analysis (linear or non-linear classifiers that use cross validation) in order to validate their cluster classification method.

REVIEWER 2

Advances the Field (Required)

It is not yet sufficiently clear if the manuscript advances the field. More details are needed.

Comments to the Authors (Required)

Lowe and Schall deploy a novel clustering approach, consensus clustering, on the issue of functional classification of the responses of frontal eye field neurons during a memory-guided saccade task. Historically, only a few classes have been described (e.g. visual, visuomovement, movement) in spite of the fact that a great deal of heterogeneity is evident among neurons classified into the same category. Clearly, arriving at a more principled classification scheme will be needed in order to understand the contribution the FEF makes to its synaptic targets and to behavior, and so this work addresses an important problem, not only for the FEF, but for the classification of neurons in any structure.

Similar to the comments raised by the first two reviewers, I have a couple of important concerns regarding the analysis and presentation of the data.

1. Similar to reviewer 2, I was surprised at the marked lack of explicit quantitative analyses to describe and validate the clustering approach. In the rebuttal, the authors state that they have addressed this issue, but it is not terribly evidence. It would be extremely valuable to, for example, provide an alternative statistical validation of a particular cluster subtype (e.g. k-means clustering, etc) to illustrate clearly that the c.c. approach arrives at subclasses that clearly differ in a qualitative manner (e.g. separate modes) rather than just a quantitative one.

2. One important challenge that exists in any attempt to optimally identify qualitatively different functional subsets of neurons with extracellular recordings is the inevitable variation in the quality of single-neuron waveform isolation. Depending on the electrode impedance, cell body size, and other factors, some isolated waveforms may be more or less contaminated with the spikes from other neurons, or have spike trains that are undersampled. In both cases, the SDFs will be distorted in different, but not random, ways depending on when other neurons fire. These effects in and of themselves, it seems, may create distinct classes of response profiles. The authors should address this, and discuss how they can address it, if at all.
